# Anticoagulant Therapy in Elderly Hospitalized Patients with Atrial Fibrillation: A Critical Appraisal of Data from the Italian REPOSI Registry

**DOI:** 10.3390/jcm15093265

**Published:** 2026-04-24

**Authors:** Silvia Accordino, Valeria Savojardo, Gabriele Ghigliazza, Alessandro Nobili, Mauro Tettamanti, Sara Ratti, Silvia Cantiero, Pier Mannuccio Mannucci, Ciro Canetta, on behalf of the REPOSI Investigators

**Affiliations:** 1 High Care Internal Medicine Unit, Foundation IRCCS Ca’ Granda Ospedale Maggiore Policlinico of Milan, 20122 Milan, Italy; 2 Department of Health Policy, Istituto di Ricerche Farmacologiche Mario Negri IRCCS, 20156 Milan, Italy; 3 Department of Clinical Research, University of Bern, 3012 Bern, Switzerland; 4 Department of Translational Medicine, University of Ferrara, 44121 Ferrara, Italy; 5 Angelo Bianchi Bonomi Haemophilia and Thrombosis Centre, Foundation IRCCS Ca’ Granda Ospedale Maggiore Policlinico of Milan, 20122 Milan, Italy

**Keywords:** atrial fibrillation, anticoagulant therapy, elderly complex patients, observational registry, data quality

## Abstract

**Background/Objectives**: Atrial fibrillation (AF) is highly prevalent among older adults and is associated with increased thromboembolic risk. Although anticoagulant therapy (AC) is strongly recommended, its use in elderly and multimorbid patients remains suboptimal. This study aimed to describe long-term trends in AC prescribing patterns among hospitalized older patients with AF. **Methods**: We conducted a retrospective observational analysis using data from the Italian REPOSI registry, including patients aged ≥65 years hospitalized with AF between 2010 and 2023. AC at admission and discharge was analyzed, including vitamin K antagonists (VKAs), direct oral anticoagulants (DOACs), and antiplatelet agents. Temporal trends, admission-to-discharge treatment changes, and patient characteristics associated with therapy modification were assessed descriptively. **Results**: The study included 2061 AF patients, characterized by multimorbidity and high thromboembolic risk. A marked shift from VKAs to DOACs was observed over time. However, a substantial proportion of cases remained without AC or received only antiplatelet therapy at both admission and discharge, with untreated individuals being generally older and more clinically complex. DOAC use increased steadily but showed a slight decline at discharge after 2020. Clinical variables available in the registry only partially explained AC changes during hospitalization. **Conclusions**: Despite increasing adoption of DOACs, AC underuse remains frequent among elderly hospitalized patients with AF. These real-world data highlight persistent challenges in AC management in complex older adults and underscore the need for more comprehensive clinical information and data-driven tools to support individualized therapeutic decision-making.

## 1. Introduction

Atrial fibrillation (AF) is a chronic and complex condition and the most common cardiac rhythm disorder in older adults. Its clinical relevance stems from the approximately five-fold increased risk of stroke and systemic thromboembolic events, which contribute to disability, mortality, and impaired quality of life. Effective thromboprophylaxis is therefore central in AF management.

Current guidelines recommend anticoagulant therapy (AC) for nearly all patients with AF, except those with a low thrombotic risk as assessed by the CHA_2_DS_2_-VA, which excludes female sex from the traditional CHA_2_DS_2_-VASc algorithm (Congestive heart failure, Hypertension, Age ≥ 75, Diabetes, Stroke/TIA/systemic embolism, Vascular disease, Age 65–74, Sex) [1].

AC options include heparin and its derivatives as well as oral anticoagulants (OACs), namely vitamin K antagonists (VKAs) and direct oral anticoagulants (DOACs). In nonvalvular AF (NVAF), DOACs—dabigatran, rivaroxaban, apixaban, and edoxaban—have demonstrated non-inferior efficacy to VKAs and have largely replaced them due to predictable pharmacokinetics, reduced bleeding risk, and the absence of routine monitoring requirements.

Despite DOAC use surpassing VKA use globally by 2017, one-quarter of AC-eligible AF patients remained untreated, and nearly one-tenth received only antiplatelet (AP) monotherapy [2], even though DOACs show superior effectiveness and safety compared with warfarin in frail older adults. Switching VKAs to DOACs in elderly patients remains a complex clinical decision [3], and among DOACs, apixaban appears to offer the most favorable safety profile without compromising protection against stroke or systemic thromboembolism [4,5].

Dose adjustments for DOAC depend on chronic kidney disease, older age, very low body weight, drug–drug interactions, high gastrointestinal bleeding risk, and severe hepatic impairment [1,6]. Nevertheless, older age, cognitive impairment, frailty, and multimorbidity remain independently associated with lower AC prescription rates [7].

In this context, bleeding risk assessment has traditionally relied on scores such as HAS-BLED (Hypertension, Abnormal Renal/Liver Function, Stroke, Bleeding History or Predisposition, Labile INR, Elderly, Drugs/Alcohol Concomitantly). However, guidelines do not endorse a specific tool and instead emphasize the identification of modifiable bleeding risk factors when prescribing or dosing AC [1,8].

Despite these recommendations, suboptimal prescribing persists, driven by therapeutic inertia and clinicians, particularly in elderly, frail, or highly complex patients [9], who are often underrepresented in pivotal randomized trials exploring drug safety and efficacy.

Real-world data from registries can provide large-scale insights into patient characteristics, prescribing behaviors, and clinical outcomes. Since 2008, the Italian REPOSI (REgistro POliterapie SIMI) multicentre observational registry has collected clinical and pharmacological data on hospitalized patients aged ≥65 years, generating a dataset of nearly 11,000 older adults and enabling extensive research on multimorbidity and polypharmacy [10]. Previous REPOSI analyses (2014–2019) showed that approximately half of older AF patients were not receiving AC therapy at admission or discharge, often without clearly documented reasons and particularly among those with multiple chronic conditions [11].

The present study expands on prior REPOSI work by examining AC prescribing patterns in elderly hospitalized AF patients over 14 years (2010–2023), spanning the full introduction and adoption of all four DOACs in Italy and including the post-pandemic years. We also describe admission-to-discharge therapy transitions and conduct exploratory analyses to identify patient characteristics potentially associated with in-hospital treatment modification.

## 2. Materials and Methods

This retrospective study analyzed a cohort of older patients with a diagnosis of AF enrolled in REPOSI, a multicentre registry conducted by the Italian Society of Internal Medicine (SIMI), IRCCS Ca’ Granda Maggiore Policlinico Hospital Foundation, and the Mario Negri Institute for Pharmacological Research. Established in 2008, REPOSI collects clinical and therapeutic information on hospitalized patients aged ≥65 years admitted to internal medicine or geriatric units. The registry is approved by the Ethics Committee of all participating centers.

Patients were eligible for inclusion if they:

- were admitted to one of the participating internal medicine or geriatric wards during the four predefined index weeks selected each season per year;
- were aged ≥65 years;
- provided written informed consent.

Data were recorded in electronic case report forms. They included socio-demographic variables, such as age and living arrangements, along with a few laboratory findings, such as hemoglobin, platelet counts, INR, and the glomerular filtration rate (GFR) calculated by applying the Cockcroft-Gault formula (kidney disease was defined as GFR < 60 mL/min); cognitive status assessed by the Short-Blessed-Test (SBT); performance in activities of daily living assessed before hospital admission by the Barthel Index (BI); severity and comorbidity index, assessed by the Cumulative-Illness-Rating-Scale, CIRS-s and CIRS-c, respectively.

Diagnoses were coded according to the International Classification of Diseases—9th Revision (ICD-9). Patients aged ≥65 years with AF identified between 2010 and 2023 through the ICD-9 code 427.3, whether recorded as the primary admission diagnosis, a comorbidity, or a reason for therapy, were included. AF status was clinician-entered in the electronic case report form based on medical history, clinical documentation, and hospital records. Patients with missing therapeutic data and those who died or were transferred during hospitalization were excluded, as discharge prescriptions were unavailable.

Therapies at admission reflected home medication regimens, whereas therapies at discharge represented prescriptions at the end of the hospital stay.

Drugs were classified according to the Anatomical Therapeutic Chemical (ATC) System as follows:

- Vitamin K antagonists (VKA): warfarin (B01AA03), acenocoumarol (B01AA07);
- Direct oral anticoagulants (DOAC): dabigatran (B01AE07), rivaroxaban (B01AF01), apixaban (B01AF02), edoxaban (B01AF03);
- Other anticoagulants: heparin group (B01AB), fondaparinux (B01AX05);
- Platelet aggregation inhibitors (B01AC).

Thromboembolic risk was assessed by the CHA_2_DS_2_-VASc score, consistent with the scoring system applied during the enrolment years. Clinical events during hospitalization and post-discharge outcomes were not analyzed due to incomplete data, particularly during the COVID-19 pandemic. Given the limited availability of information on drug dosages, contraindications, and clinical decision-making, the study focused on descriptive analyses of AC prescribing patterns. Temporal trends from 2010 to 2023 were examined, with detailed admission-to-discharge comparisons from 2016 onward, when all four DOACs were available in Italy.

### Statistical Analysis

Descriptive statistics were used to summarize the clinical characteristics. Categorical variables were reported as absolute frequencies and percentages, and continuous variables as medians with interquartile range (IQR). Graphical representations were used to illustrate changes in antithrombotic prophylaxis over time.

Exploratory logistic regression models were performed to investigate potential predictors of treatment modification, particularly DOAC initiation at discharge among patients admitted without AC or on VKA. Univariate models were first estimated, followed by models adjusted for age and sex. A fully adjusted multivariate logistic regression model was then constructed, including all covariates simultaneously in a single step, based on clinical plausibility and their role as potential confounders. Covariates included in the multivariate models were sex, age, weight, CIRS severity index, Barthel index, CHA_2_DS_2_-VASC score, hemoglobin, platelet count, INR, and estimated GFR. For each model, odds ratios (ORs), 95% confidence intervals (95%CIs), and *p*-values were reported. Missing data were handled through multiple imputation using chained equations (MICE), with 100 imputed datasets generated. The proportion of missing data per variable was as follows: Barthel Index 15.6%, body weight 9.9%, eGFR 10.8%, INR 6.8%, hemoglobin 0.5%, platelet count 0.9%, and CIRS Illness Severity Index 0.1%. All variables included in the regression models were imputed, except sex, age, and CHA_2_DS_2_-VASc score, which were fully observed and used as predictors in the imputation model. This approach was chosen to preserve statistical power given the moderate sample size. Convergence was assessed by examining the stability of variable means across iterations; no systematic drift was observed, indicating adequate convergence of the chained equations algorithm. Given the observational nature of the registry and the extent of missingness, these analyses are considered exploratory.

All analyses were carried out using Stata/IC v15.1 (StataCorp, College Station, TX, USA), and all graphs were created using Microsoft Excel v2021.

## 3. Results

**Figure 1:**
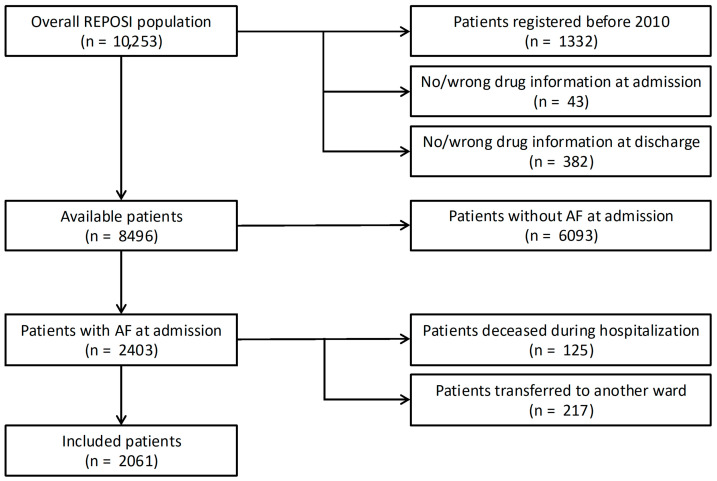
Flow-chart of the population selection.

Of the 10,253 patients enrolled in the REPOSI register since 2008, 1757 were excluded because they were admitted before 2010 or had missing therapeutic information. Among the remaining 8496 patients, 2403 had AF at admission; of these, 125 died during hospitalization, and 217 were transferred to other wards. After these exclusions, the final cohort consisted of 2061 AF patients [Figure 1].

Baseline characteristics remained broadly stable over the study period. Patients were consistently elderly, multimorbid, and exhibited a high thromboembolic risk [Table 1].

**Figure 2:**
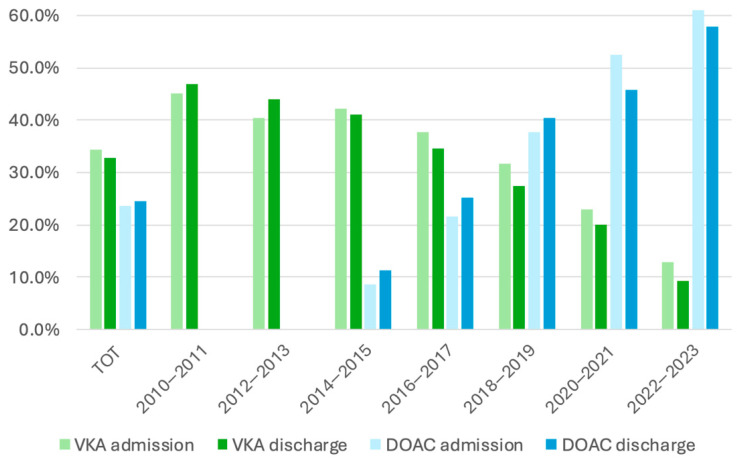
VKA and DOAC at admission and discharge by admission year (2010–2023).

Figure 2 illustrates the temporal evolution of AC prescription. A clear shift from VKAs to DOACs was observed. Until 2014, VKA prescriptions at discharge increased; from 2014 onwards, VKA use progressively declined, while DOAC prescriptions rose steadily. After 2020, a slight decrease in DOAC initiation at discharge was noted, paralleling a similar decline in VKA use [Figure 2]. Given the small sample size and increased missingness during the pandemic years, this late-period fluctuation should be interpreted as a descriptive observation rather than a definitive trend.

Between 2016 and 2023, among 1152 patients admitted during the period in which all four DOACs were available, 40.1% were DOAC users, 13.5% received no AC, and 11.9% received only AP therapy. At discharge, 51.3% of patients admitted without AC and 51.8% of those receiving only AP remained untreated or on AP monotherapy. Among DOACs, dabigatran and edoxaban were more frequently changed during hospitalization compared with rivaroxaban and apixaban [Table 2].

Patients discharged without AC or with only AP were generally older, whereas other clinical characteristics showed minimal variation across treatment groups [Table 3].

From 2010 to 2023, CIRS severity and INR were associated with changes in AC therapy from admission to discharge, likely reflecting in-hospital switching from VKAs to DOACs among less comorbid patients. When restricting analyses to 2016–2023 and applying the same multiple imputation strategy, only hemoglobin values were statistically significant predictors of AC modification [Supplementary Materials Table S1a,b].

In the same period, exploratory analyses of patients initiating DOACs during hospitalization or at discharge suggested that lower CIRS severity and higher eGFR might be associated with DOAC initiation. However, these associations did not reach statistical significance and lacked clear clinical relevance, particularly regarding age, weight, and comorbidity burden in patients switched from VKAs to DOACs [Supplementary Materials Table S2a,b].

## 4. Discussion

Anticoagulant management in older adults with AF remains a major clinical challenge, particularly in the context of multimorbidity, functional impairment, and acute medical conditions. In this cohort of hospitalized patients aged ≥65 years, we observed substantial temporal changes in prescribing patterns over more than a decade, with a clear and progressive shift from VKAs to DOACs. The long observation window of the REPOSI registry allowed us to capture the full trajectory of DOAC introduction and adoption in Italy, documenting how clinical practice gradually incorporated these agents into routine care.

AC prescribing appropriateness encompasses several dimensions, including the absence of treatment despite indication, the use of AC in the presence of contraindications, or the prescription of DOACs or VKAs under conditions or at dosages inconsistent with guideline recommendations. Although hospitalization should, in principle, facilitate optimization of AC therapy, a substantial proportion of older AF patients continue to receive no AC or potentially suboptimal regimens [12].

In this cohort, thromboembolic risk was predominantly driven by advanced age and multimorbidity; however, despite transition to DOACs, a considerable proportion of these high-risk patients remained untreated at both admission and discharge. This finding is consistent with previous REPOSI analyses [11] and with global data indicating persistent underuse of anticoagulation in older adults with AF [2].

The slight decline in DOAC initiation after 2020 should be interpreted with caution; recruitment during the COVID-19 pandemic was markedly reduced, and missing data were more frequent, limiting the stability of trend estimates. This pattern should therefore be viewed as descriptive rather than indicative of a true reversal.

The clinical complexity of this population is substantial, since comorbidity burden and polypharmacy may adversely influence decision-making. Untreated individuals tended to be older and more clinically complex, suggesting that concerns about bleeding risk, frailty, or competing clinical priorities may continue to influence prescribing decisions. The persistence of AP monotherapy in a subset of patients may further reflect the difficulty of balancing thromboembolic and hemorrhagic risks in multimorbid older adults, particularly when clinical information is incomplete or rapidly evolving.

Notably, the 125 patients who died during hospitalization, excluded from discharge analyses, likely represent an even more vulnerable subgroup.

High rates of multimorbidity, acute reasons for hospital admission, and functional impairment underscore the importance of optimal medical therapy in frail older adults, even given the role of AF as a trigger for acute decompensation [13].

Patterns of therapy modification during hospitalization also provide insight into clinicians’ decision-making. Among patients admitted without AC or on AP therapy alone, approximately half remained untreated at discharge, indicating that hospitalization did not consistently lead to optimization of thromboprophylaxis.

However, interpretation of registry-based analyses requires caution: guideline adherence cannot be inferred solely from CHA_2_DS_2_-VASc risk categories, nor can overtreatment be defined exclusively by the concomitant use of AC and AP therapy [14]. Regression models may identify predictors of AC use or discontinuation, and mortality analyses may incorporate treatment groups and covariates such as comorbidity or frailty, but the impact of AC on outcomes and the full spectrum of appropriateness cannot be robustly assessed without detailed clinical information.

Previous nationwide studies have combined multiple administrative and clinical databases to improve completeness, yet they share limitations similar to those of the present analysis, including reliance on diagnostic codes and incomplete documentation of clinical reasoning [15,16,17].

Registries offer valuable opportunities for large-scale pharmaco-epidemiological research, reducing selection bias and providing insights into prescribing behaviors in populations underrepresented in randomized trials, such as frail older adults [18], but they also face inherent limitations: missing data, inconsistent coding, non-standardized diagnostic criteria, and lack of detailed information on contraindications, drug dosages, bleeding history, frailty indices, and clinical trajectories.

Interpretation of antiplatelet therapy patterns is also limited by the absence of detailed information on coronary or vascular indications. Because AP therapy is not a substitute for AC in eligible AF patients, some individuals receiving AP monotherapy or combination therapy may have had valid indications (e.g., recent PCI or acute coronary syndrome). Therefore, these categories cannot be assumed to reflect inappropriate management.

Data entry inconsistencies, incomplete documentation, and the absence of systematic validation further contribute to missing or inaccurate information [19]. As a result, the longitudinal perspective of REPOSI reveals important trends and describes prescribing patterns, but cannot determine appropriateness at the individual level, with the risk of overestimating the discrepancies between actual prescribing practices and the guidelines’ recommendations.

A major limitation of large registries remains the trade-off between sample size, data quality, and missing information. From a future perspective, the integration of Artificial Intelligence and Machine Learning into Electronic Health Records (EHR) has the potential to mitigate some of these gaps [20].

Our results highlight the need for more complete and standardized clinical information to enable robust assessment of AC appropriateness in future research and to inform precision-medicine approaches. The REPOSI experience offers a valuable foundation for data-driven innovations and underscores the importance of interoperable, accurate, and automated data-collection systems capable of supporting evaluations of therapy quality to improve data completeness and interpretability. Large multicentre registries could enable the creation of appropriately powered cohorts suitable for AI-based analyses, improving prediction accuracy and supporting long-term monitoring of complex conditions such as AF, supporting clinical decision-making and quality of care with individualized risk estimates and decision-support algorithms that balance thromboembolic and bleeding risks for more personalized treatment strategies.

## 5. Limitations

This study has several limitations inherent to the use of routinely collected registry data. REPOSI is a non-interventional observational registry with broad generalizability, not specifically designed to evaluate AC therapies.

This register does not systematically capture key variables required to assess AC appropriateness, including drug dosages, drug–drug interactions, specific contraindications, INR variability, prior bleeding events, comprehensive frailty indices, and fall history. Data for risk stratification—such as evolving renal function, history of falls, complete and reliable diagnostic histories, reasons for concomitant AP therapy, and recent acute conditions—were not uniformly available.

Kidney disease was defined using a single eGFR threshold, without longitudinal renal function data, and the current use of the CHA_2_DS_2_-VA score, which excludes female sex, may partially affect comparability with studies adopting the traditional CHA_2_DS_2_-VASc score.

Additional challenges concern ICD-9 diagnostic codes, whose accuracy varies across centers and over time. Coding inconsistencies may lead to under- or over-reporting of comorbidities, affecting risk score calculations, dose-reduction criteria, and identification of contraindications, such as life expectancy ≤ 1 year, active malignancies, dialysis status, severe mitral stenosis or implanted mechanical heart valves, rheumatic disease, antiphospholipid syndrome, or left ventricular thrombosis.

These limitations restrict the ability to determine the appropriateness of prescribing at the individual level and limit the interpretability of treatment patterns.

Finally, the reduced sample size and increased missingness during the COVID-19 pandemic limit the stability of trend estimates for the most recent years, and patients who died during hospitalization or were transferred to other wards were excluded from discharge analyses, potentially introducing selection bias. Missing data required multiple imputation, and regression analyses should be interpreted as exploratory.

## 6. Conclusions

In this large cohort of elderly hospitalized patients with AF, we observed substantial temporal changes in anticoagulant prescribing, including a marked shift from VKAs to DOACs. Despite this evolution, a significant proportion of high-risk patients remained untreated or received antiplatelet monotherapy at both admission and discharge. These findings highlight persistent challenges in translating guideline recommendations into practice in multimorbid older adults and underscore the complexity of anticoagulation management in this population.

Overall, the results emphasize the need for more comprehensive and structured clinical information to support individualized therapeutic decision-making. Improving data completeness and integrating decision-support tools into routine care may help to promote more consistent application of evidence-based strategies in older complex patients with AF.

## Figures and Tables

**Table 1:** Clinical characteristics at admission (2010–2023).

	Total	2010–2011	2012–2013	2014–2015	2016–2017	2018–2019	2020–2021	2022–2023
	N = 2061	N = 278	N = 321	N = 310	N = 358	N = 385	N = 105	N = 304
**Age 65–74**	352 (17.1%)	51 (18.3%)	58 (18.1%)	59 (19.0%)	49 (13.7%)	64 (16.6%)	22 (21.0%)	49 (16.1%)
**Age 75–84**	939 (45.6%)	141 (50.7%)	152 (47.4%)	164 (52.9%)	161 (45.0%)	156 (40.5%)	45 (42.9%)	120 (39.5%)
**Age ≥ 85**	770 (37.4%)	86 (30.9%)	111 (34.6%)	87 (28.1%)	148 (41.3%)	165 (42.9%)	38 (36.2%)	135 (44.4%)
**Male**	1003 (48.7%)	128 (46.0%)	155 (48.3%)	157 (50.6%)	168 (46.9%)	177 (46.0%)	61 (58.1%)	157 (51.6%)
**At least one previous hospitalization**	866 (42.0%)	109 (39.2%)	125 (38.9%)	136 (43.9%)	154 (43.1%)	158 (41.0%)	49 (46.7%)	135 (44.4%)
**CIRS severity**	1.7 (1.5–1.9)	1.7 (1.5–1.8)	1.7 (1.5–2.0)	1.7 (1.5–2.0)	1.7 (1.5–1.9)	1.7 (1.5–1.8)	1.8 (1.5–1.9)	1.7 (1.5–1.9)
**CIRS comorbidity**	3.0 (2.0–5.0)	3.0 (2.0–4.0)	3.0 (2.0–5.0)	3.0 (2.0–5.0)	3.0 (2.0–4.0)	3.0 (2.0–5.0)	3.0 (2.0–5.0)	3.0 (2.0–4.0)
**Dementia from Profound Index**	853 (45.5%)	140 (50.7%)	161 (52.3%)	104 (37.8%)	147 (45.0%)	133 (41.8%)	39 (41.5%)	129 (46.4%)
**Barthel**	87.0 (63.5–100.0)	NA	90.0 (63.0–100.0)	90.0 (70.0–100.0)	85.0 (65.0–99.0)	83.0 (58.0–97.0)	86.0 (61.0–98.0)	87.0 (64.0–100.0)
**Liver Disease**	45 (2.2%)	9 (3.2%)	6 (1.9%)	9 (2.9%)	5 (1.4%)	10 (2.6%)	1 (1.0%)	5 (1.6%)
**Kidney Disease**	648 (31.4%)	71 (25.5%)	78 (24.3%)	106 (34.2%)	127 (35.5%)	126 (32.7%)	35 (33.3%)	105 (34.5%)
**Stroke/TIA**	266 (12.9%)	33 (11.9%)	43 (13.4%)	44 (14.2%)	46 (12.8%)	43 (11.2%)	16 (15.2%)	41 (13.5%)
**Heart Failure**	793 (38.5%)	100 (36.0%)	106 (33.0%)	118 (38.1%)	141 (39.4%)	154 (40.0%)	41 (39.0%)	133 (43.8%)
**Diabetes**	590 (28.6%)	73 (26.3%)	95 (29.6%)	82 (26.5%)	114 (31.8%)	115 (29.9%)	35 (33.3%)	76 (25.0%)
**Ischemic Heart Disease**	578 (28.0%)	78 (28.1%)	100 (31.2%)	90 (29.0%)	108 (30.2%)	107 (27.8%)	33 (31.4%)	62 (20.4%)
**Atherosclerosis**	836 (40.6%)	106 (38.1%)	139 (43.3%)	135 (43.5%)	158 (44.1%)	158 (41.0%)	47 (44.8%)	93 (30.6%)
**CHA_2_DS_2_VASc**	4.0 (4.0–5.0)	4.0 (4.0–5.0)	4.0 (4.0–5.0)	4.0 (4.0–5.0)	4.0 (4.0–5.0)	4.0 (4.0–5.0)	4.0 (3.0–6.0)	4.0 (3.0–6.0)
**Hb (g/dL)**	11.8 (10.1–13.3)	12.4 (10.7–13.8)	12.1 (10.4–13.6)	11.8 (10.1–13.5)	11.6 (10.2–13.0)	11.8 (10.1–13.1)	11.2 (9.8–12.9)	11.5 (9.6–12.9)
**Platelets (10^9^/mmc)**	211.0 (168.0–272.0)	215.0 (171.0–274.5)	212.0 (171.0–265.0)	214.0 (173.0–278.0)	215.0 (173.0–272.0)	205.0 (163.0–271.0)	202.0 (168.0–276.0)	211.0 (163.0–270.0)
**INR**	1.3 (1.1–2.0)	1.3 (1.1–2.4)	1.2 (1.1–2.2)	1.3 (1.0–2.1)	1.3 (1.1–2.3)	1.3 (1.1–1.9)	1.2 (1.1–1.7)	1.2 (1.1–1.5)
**Creatinine (mg/dL)**	1.1 (0.8–1.5)	1.1 (1.1–2.4)	1.1 (0.8–1.5)	1.1 (0.8–1.5)	1.1 (0.9–1.5)	1.1 (0.9–1.5)	1.1 (0.9–1.4)	1.1 (0.9–1.6)

CIRS (Cumulative-Illness-Rating-Scale); TIA (Transient Ischemic Attack); CHA2DS2VASc (Congestive heart failure, Hypertension, Age ≥ 75, Diabetes, Stroke/TIA/systemic embolism, Vascular disease, Age 65–74, Sex).

**Table 2:** Changes in therapies comparing admission and discharge (2016–2023).

	Therapy at Discharge						
	No AC	AC (No DOAC)	Dabigatran	Rivaroxaban	Apixaban	Edoxaban	Only AP
	N = 159	N = 436	N = 59	N = 131	N = 207	N = 73	N = 87
**Therapy at admission**							
**No AC** **N = 156**	80 (51.3%)	28 (17.9%)	3 (1.9%)	9 (5.8%)	21 (13.5%)	10 (6.4%)	5 (3.2%)
**AC (no DOAC)** **N = 397**	29 (7.3%)	327 (82.4%)	5 (1.2%)	9 (2.3%)	16 (4.0%)	3 (0.8%)	8 (2.0%)
**Dabigatran** **N = 68**	4 (5.9%)	7 (10.3%)	50 (73.6%)	0	4 (5.9%)	2 (2.9%)	1 (1.4%)
**Rivaroxaban** **N = 134**	13 (9.7%)	7 (5.2%)	1 (0.8%)	111 (82.8%)	2 (1.5%)	0	0
**Apixaban** **N = 187**	15 (8.0%)	17 (9.1%)	0	0	152 (81.3%)	1 (0.5%)	2 (1.1%)
**Edoxaban** **N = 73**	9 (12.3%)	8 (11.0%)	0	0	3 (4.1%)	53 (72.6%)	0
**Only AP** **N = 137**	9 (6.6%)	42 (30.7%)	0	2 (1.5%)	9 (6.5%)	4 (2.9%)	71 (51.8%)

AC (Anticoagulants); DOAC (Direct anticoagulants); AP (Antiplatelets).

**Table 3:** Clinical characteristics according to drugs at discharge (2016–2023).

	No AC	DOAC	AC (No DOAC)	Only AP	AC + AP
	N = 159	N = 437	N = 376	N = 87	N = 93
**Age 65–74**	31 (19.5%)	68 (15.6%)	51 (13.6%)	13 (14.9%)	21 (22.6%)
**Age 75–84**	51 (32.1%)	189 (43.2%)	170 (45.2%)	35 (40.2%)	37 (39.8%)
**Age ≥ 85**	77 (48.4%)	180 (41.2%)	155 (41.2%)	39 (44.8%)	35 (37.6%)
**Male**	71 (44.7%)	214 (49.0%)	181 (48.1%)	39 (44.8%)	58 (62.4%)
**Weight**	69.0 (60.0–78.0)	71.0 (61.7–82.0)	70.0 (61.0–80.0)	72.0 (60.0–80.0)	70.5 (63.0–82.0)
**BMI**	24.3 (22.4–27.5)	25.6 (23.3–29.4)	25.6 (23.0–29.4)	26.3 (22.8–29.4)	26.0 (23.4–30.1)
**At least one previous hospitalization**	70 (44.0%)	181 (41.4%)	163 (43.4%)	33 (37.9%)	49 (53.3%)
**CIRS severity**	1.8 (1.5–2.0)	1.7 (1.5–1.8)	1.8 (1.5–2.0)	1.7 (1.5–2.0)	1.8 (1.6–2.0)
**CIRS comorbidity**	3.0 (2.0–5.0)	3.0 (2.0–4.0)	3.0 (2.0–5.0)	3.0 (2.0–4.0)	4.0 (2.0–5.0)
**Dementia from Profound Index**	58 (46.8%)	150 (39.5%)	166 (47.7%)	40 (47.6%)	34 (42.0%)
**Short Blessed**	8.0 (4.0–14.0)	6.0 (2.0–12.0)	8.0 (4.0–14.0)	7.0 (4.0–14.0)	6.0 (2.0–12.0)
**Barthel**	82.0 (57.0–98.0)	88.0 (71.0–100.0)	83.0 (56.0–98.0)	81.0 (53.0–97.0)	85.0 (57.0–98.0)
**Hb (g/dL)**	11.2 (9.2–12.8)	12.0 (10.6–13.3)	11.3 (9.6–12.9)	11.6 (10.4–12.9)	11.3 (10.1–13.0)
**PLT (10^9^/mmc)**	195.0 (152.0–262.0)	215.0 (171.0–271.5)	211.0 (169.0–274.0)	204.0 (164.0–250.0)	216.0 (167.0–278.0)
**INR**	1.2 (1.0–1.4)	1.2 (1.1–1.5)	1.9 (1.2–2.6)	1.1 (1.0–1.2)	1.3 (1.1–1.9)
**Creatinine (mg/dL)**	1.1 (0.9–1.6)	1.0 (0.8–1.3)	1.1 (0.9–1.6)	1.2 (0.9–1.7)	1.2 (0.9–1.8)

CIRS (Cumulative-Illness-Rating-Scale); AC (Anticoagulants); DOAC (Direct anticoagulants); AP (Antiplatelets).

## Data Availability

The REPOSI data are available after a reasonable request to the study team, upon receipt of a project compatible with the register’s database.

## Supplementary Materials

The following supporting information can be downloaded at: https://www.mdpi.com/article/10.3390/jcm15093265/s1, Table S1a: Multiple Imputation for the association with change in drug therapy at discharge (2010–2023). Table S1b: Multiple Imputation for the association with change in drug therapy at discharge (2016–2023). Table S2a: Change in drug therapy at discharge (2016–2023): No DOACs at admission to DOACs at discharge. Table S2b: Change in drug therapy at discharge (2016–2023): VKAs at admission to DOACs at discharge.

## Abbreviations

The following abbreviations are used in this manuscript:

AF	Atrial fibrillation
NVAF	Non valvular atrial fibrillation
AC	Anticoagulant
OAC	Oral anticoagulant
VKA	Vitamin K antagonists
DOAC	Direct anticoagulants
AP	Antiplatelets
SEE	Systemic thromboembolic events

## Appendix A. Investigators and Co-Authors of the REPOSI (Registro POliterapie SIMI, Società Italiana Di Medicina Interna) Study Group

Steering Committee: Pier Mannuccio Mannucci (Chair) (Fondazione IRCCS Cà Granda Ospedale Maggiore Policlinico, Milano), Alessandro Nobili (co-chair) (Istituto di Ricerche Farmacologiche Mario Negri IRCCS, Milano), Nicola Montano (Presidente SIMI), Giorgio Sesti (Past-President-SIMI), Antonello Pietrangelo (Direttore CRIS-SIMI), Antonio De Vincentis (Policlinico Universitario Campus Bio-Medico, Roma), Alessandra Marengoni (Spedali Civili di Brescia, Brescia), Mauro Tettamanti, Luca Pasina, Carlotta Franchi (Istituto di Ricerche Farmacologiche Mario Negri IRCCS, Milano).
Clinical Data Monitoring and Revision: Francesca Orsini, Massimo Cartabia, Gabriella Miglio (Istituto di Ricerche Farmacologiche Mario Negri IRCCS, Milano).
Database Management and Statistics: Alessia Antonella Galbussera, Sara Mandelli, Ilaria Ardoino, Alessio Novella, Sara Ratti, Enrico Nicolis (Istituto di Ricerche Farmacologiche Mario Negri IRCCS, Milano).
Investigators:
Domenico Prisco, Elena Silvestri, Giacomo Emmi, Irene Mattioli, Matteo Mazzetti, Edoardo Biancalana (Azienda Ospedaliero Universitaria Careggi Firenze, SOD Medicina Interna Interdisciplinare);
Gianni Biolo, Giacomo Bartelloni, Ilaria Martini (Azienda Sanitaria Universitaria Integrata di Trieste, Clinica Medica Generale e Terapia Medica);
Matteo Pirro, Graziana Lupattelli, Vanessa Bianconi, Riccardo Alcidi, Alessia Giotta, Massimo R. Mannarino (Azienda Ospedaliera Santa Maria della Misericordia, Perugia, Medicina Interna, Angiologia Malattie da Arteriosclerosi);
Domenico Girelli, Fabiana Busti, Giacomo Marchi (Azienda Ospedaliera Universitaria Integrata di Verona, Verona, Medicina d’Urgenza);
Nicola Veronese, Mario Barbagallo, Ligia Dominguez, Vincenza Beneduce, Federica Cacioppo, Stefano Ciriminna, Andrea Asaro, Chiara Giannettino, Claudia Canizzo, Giovanna Ottavia Plano, Anna Fazzari, Alessandra Lo Nigro, Ilenia Imbraguglio, Sofia Contorno (Azienda Ospedaliera Universitaria Policlinico Giaccone Policlinico di Palermo, Palermo, Unità Operativa di Geriatria e Lungodegenza);
Salvatore Corrao, Giuseppe Natoli, Salvatore Mularo, Valentina Orlando, Giacomo Corrao, Fabio Falcone, Salvatore Scibetta, Luigi Mirarchi, Simona Amodeo, Brenda Bongiorno (A.R.N.A.S. Civico, Di Cristina, Benfratelli, Palermo, UOC Medicina Interna ad Indirizzo Geriatrico-Riabilitativo);
Marco Zoli, Maria Laura Matacena, Giuseppe Orio, Eleonora Magnolfi, Giovanni Serafini, Mattia Brunori, Ilaria Lazzari, Angelo Simili (Azienda Ospedaliera Universitaria Policlinico S. Orsola-Malpighi, Bologna, Unità Operativa di Medicina Interna Zoli);
Tiziano Lucchi, Paola Nicolini, Gabriele Ghidini, Miriana Martelengo, Maddalena Fabrizi, Giulia Vigani, Arturo Cerizza, Rita Deda, Miriam Zappa, Benedetta Carrelli (Fondazione IRCCS Cà Granda Ospedale Maggiore Policlinico, Milano, Geriatria);
Antonio Di Sabatino, Emanuela Miceli, Marco Vincenzo Lenti, Lavinia Pitotti, Valentina Antoci, Ginevra Cambiè, Costanza Caccia Dominioni (IRCCS Policlinico San Matteo di Pavia, Pavia, Clinica Medica I, Reparto 11);
Roberto Pontremoli, Valentina Beccati, Giulia Nobili, Giovanna Leoncini, Jacopo Alberto, Federico Cattaneo (IRCCS Azienda Ospedaliera Universitaria San Martino-IST di Genova, Genova, Clinica di Medicina Interna 2);
Francesco Cipollone, Maria Teresa Guagnano, Ilaria Rossi, Damiano D’Ardes, Alessia Cipollone, Riccardo Mattia Ricciardi, Marco Bucci, Paola Vizzarri, Artenca Shehu (Ospedale Clinicizzato SS. Annunziata, Chieti, Clinica Medica);
Gerardo Mancuso, Daniela Calipari, Mosè Bartone (Ospedale Giovanni Paolo II Lamezia Terme, Catanzaro, Unità Operativa Complessa Medicina Interna);
Roberto Manetti, Marta Chiara Sircana, Maria Berria, Alessandro Delitala, Pierluigi Meloni, Silvia Masala (Cliniche San Pietro, Azienda Ospedaliera Universitaria di Sassari, S.C. Clinica Medica);
Maurizio Muscaritoli, Alessio Molfino, Antonella Giorgi, Christian Gracin, Giovanni Imbimbo, Carmen Gallicchio, Ottavio Martellucci, Antonietta Gigante, Chiara D’Elia (Policlinico Umberto I, Sapienza Università di Roma, Medicina Interna e Nutrizione Clinica Policlinico Umberto I);
Alessandra Marengoni, Thelma Geneletti, Eleonora Carlotti, Arianna Pagani, Claudia Capelli (SC Geriatria, Spedali Civili, Montichiari, Brescia);
Antonio Picardi, Umberto Vespasiani Gentilucci, Paolo Gallo, Jessica Avagliano, Francesca Terracciani (Università Campus Bio-Medico, Roma, Medicina Clinica-Epatologia);
Giuseppe Bellelli, Maurizio Corsi, Lavinia Vitali, Armida Soccali (Università degli Studi di Milano-Bicocca e Unità Operativa Complessa di Geriatria, IRCCS Fondazione S. Gerardo, Monza);
Franco Arturi, Elena Succurro, Melania Melina, Federica Giofrè, Francesca Cosentino, Delia Chiarello, Francesco Andreozzi (Università degli Studi Magna Graecia, Azienda Ospedaliera-Universitaria “Renato Dulbecco”, Catanzaro, Unità Operativa Complessa di Medicina Interna);
Antonio Brucato, Teresa De Falco, Enrica Negro, Martino Brenna, Lucia Trotta, Giovanni Lorenzo Squintani, Giacomo Iacomelli, Giulia Colazzo, Francesco Cavaleri (ASST Fatebenefratelli-Sacco, Milano, Medicina Interna);
Paolo Simioni, Irene Bertozzi, Sara Angela Malerba, Camilla Portinari, Giordano Antonello, Cecilia Fortino, Camilla Goffi, Francesca Guidolin (Azienda Ospedaliera Università di Padova, Padova, Clinica Medica I);
Roberto Manfredini, Benedetta Boari, Alfredo De Giorgi, Ruana Tiseo, Giulia Marta Viglione, Caterina Savriè (Azienda Ospedaliera-Universitaria Sant’Anna, Ferrara, Unità Operativa Clinica Medica);
Giuseppe Paolisso, Maria Rosaria Rizzo, Claudia Catalano, Irene Di Meo, Michele Cerasuolo, Nicola Pelligrino (Azienda Ospedaliera Universitaria Luigi Vanvitelli- Università della Campania Luigi Vanvitelli, Napoli, Unità Operativa Complessa di Geriatria e Medicina Interna);
Claudio Borghi, Federica Piani, Federico Ruscelli, Chiara Baldini, Giulia Boni (Medicina Interna Cardiovascolare, IRCCS Azienda Ospedaliero-Universitaria di Bologna);
Carlo Sabbà, Francesco Saverio Vella, Patrizia Suppressa, Alessio Comitangelo, Emanuele Amoruso, Carlo Custodero, Giuseppe Re, Chiara Maria Palmisano (Azienda Ospedaliero-Universitaria Consorziale Policlinico di Bari, Bari, Medicina Interna Universitaria C. Frugoni);
Luigi Fenoglio, Christian Bracco, Giulia Racca, Alessia Valentina Giraudo, Salvatore D’Agnano, Giorgia Sasia, Irene Ruocco, Floriana Mao, Alice Ferrua, Chiara Candela (Azienda Sanitaria Ospedaliera Santa Croce e Carle di Cuneo, Cuneo, S. C. Medicina Interna);
Anna Ludovica Fracanzani, Silvia Tiraboschi, Annalisa Cespiati, Giovanna Oberti, Giordano Sigon, Felice Cinque, Lucia Colavolpe, Jaqueline Currà, Francesca Alletto, Natalia Scaramellini, Simona Leoni, Alessandra Danuta Di Mauro, Gianpaolo Benzoni, Margherita Re, Laura Vivi, Valentina Scime (Fondazione IRCCS Cà Granda Ospedale Maggiore Policlinico, Milano, UOC Medicina Generale ad Indirizzo Metabolico);
Flora Peyvandi, Raffaella Rossio, Giulia Colombo, Pasquale Agosti, Erica Pagliaro (Fondazione IRCCS Cà Granda Ospedale Maggiore Policlinico, Milano, Medicina Interna 2, SC Medicina Emostasi e Trombosi);
Ciro Canetta, Valeria Savojardo, Giuliana Ceriani, Christian Folli (Fondazione IRCCS Cà Granda Ospedale Maggiore Policlinico, Milano, Medicina Interna Alta Intensità di Cure);
Fabrizio Montecucco, Cristina Michelauz, Elisa Schiavetta, Chiara Olivero, Simone Isoppo, Curzia Tortorella, Federico Carbone, Luca Liberale, Aurora Mazzocato, Carola Caserza, Anita Cennamo (IRCCS Ospedale Policlinico San Martino e Università di Genova, Genova, Clinica Medica 1, Medicina Interna e Specialità Mediche);
Nicola Lucio Liberato, Tiziana Tognin (ASST di Pavia, UOSD Medicina Interna, Ospedale di Casorate Primo, Pavia);
Francesco Purrello, Antonino Di Pino, Salvatore Piro, Maurizio Di Marco, Nicoletta Miano, Sabrina Scilletta (Ospedale Garibaldi Nesima, Catania, Unità Operativa Complessa di Medicina Interna);
Renzo Rozzini, Francesca Mazzeo, Giulia Cono, Giulia Cesaroni (Ospedale Poliambulanza, Brescia, Medicina Interna e Geriatria);
Giuseppe Montrucchio, Paolo Peasso, Edoardo Favale, Cesare Poletto, Carl Margaria, Maura Sanino, Valeria Bianchi, Beatrice Bovo, Beatrice Brusasco, Gabriele Giuliano, Marco Angeleri (Dipartimento di Scienze Mediche, Università di Torino, Città della Scienza e della Salute, Torino, Medicina Interna 2 Unità Indirizzo d’Urgenza);
Luigina Guasti, Francesca Rotunno, Luana Castiglioni, Andrea Maresca, Leonardo Campiotti, Alessandra Grossi, Roberto Davide Diprizio, Francesco Dentali, Veronica Behnke, Gloria Cuni, Elena Fedra Salati (Università degli Studi dell’Insubria, Ospedale di Circolo e Fondazione Macchi, Varese, Medicina e Geriatria);
Marco Bertolotti, Chiara Mussi, Giulia Lancellotti, Laura Orlandi, Federica Di Zio, Gabriele Luppi, Francesca D’Imprima, Chiara Mazza, Claudia Grandi, Chiara Ferrari, Ambrogio Breschi (Università di Modena e Reggio Emilia, Azienda Ospedaliero-Universitaria di Modena; Ospedale Civile di Baggiovara, Unità Operativa di Geriatria);
Angela Sciacqua, Maria Perticone, Raffaele Maio, Aleandra Scozzafava, Valentino Condoleo, Elvira Clausi, Giuseppe Armentaro, Alberto Panza, Alessia Grandinetti, Maria Rosa, Carlo Alberto Pastura, Pasquale Loiacono, Mariarosangela Scarcelli (Università Magna Graecia Policlinico Renato Dulbecco, Catanzaro, DAI Medicina Interna, Unità Operativa Geriatria);
Gianluca Moroncini, Emanuele Filippini, Devis Benfaremo (Clinica Medica, Azienda Ospedaliera- Universitaria delle Marche, Ancona);
Pietro Castellino, Luca Zanoli, Agostino Gaudio, Anastasia Xourafa, Concetta Spichetti, Serena Torre, Alfio Gennaro, Lucrezia Taibi, Francesca Ferrari (Azienda Ospedaliera Universitaria Policlinico–V. Emanuele, Catania, Dipartimento di Medicina);
Guido Moreo, Silvia Prolo, Gloria Pina, Lorenzo Magni, Sophia Ratzinger (Clinica San Carlo Casa di Cura Polispecialistica, Paderno Dugnano, Milano, Unità Operativa di Medicina Generale Emilio Bernardelli);
Alberto Ballestrero, Fabio Ferrando, Roberta Gonella, Arianna Piccardo, Andrea Bellodi, Dejvis Kalaja (Clinica Di Medicina Interna ad Indirizzo Oncologico, Azienda Ospedaliera Università San Martino di Genova);
Anna Linda Patti, Giuseppe Famularo, Patrizia Tarsitani, Tiziana Morretti, Andrea Aglitti (Azienda Ospedaliera San Camillo Forlanini, Roma, Medicina Interna II);
Marcello Giuseppe Maggio, Fulvio Lauretani, Marco Salvi, Chiara Cattabiani, Elena Bronzoni, Aida Hoxha, Beatrice Tanzi (Azienda Ospedaliero Universitaria di Parma, U.O.C Clinica Geriatrica);
Stefano Del Giacco, Davide Firinu, Giulia Costanzo, Andrea Giovanni Ledda, Matteo Benuzzi, Salvatore Chessa, Martina Bullita (Policlinico Universitario Duilio Casula, Azienda Ospedaliero-Universitaria di Cagliari, Cagliari, Medicina Interna, Allergologia ed Immunologia Clinica);
Anna Licata, Giuseppe Montalto, Filippo Alessandro Montalto, Angelo Rizzo, Silvia Como, Valentina Virzì (Azienda Ospedaliera Universitaria Policlinico Paolo Giaccone, Palermo, UOC di Medicina Interna);
Giorgio Basile, Antonino Catalano, Federica Bellone, Concetto Principato, Angelo Cocuzza, Annamaria Buda, Francesco Corica (Azienda Ospedaliera Universitaria Policlinico G. Martino, Messina, Unità Operativa di Geriatria);
Lorenzo Malatino, Benedetta Stancanelli, Valentina Terranova, Salvatore Di Marca, Rosario Di Quattro, Lara La Malfa, Rossella Caruso, Ivan Isaia, Federica Castelletti, Matteo Regolo, Clara Salmeri, Nicolas Cardaci, Alessandra Lanzafame (Azienda Ospedaliera per l’Emergenza Cannizzaro, Catania, Clinica Medica Università di Catania);
Patrizia Mecocci, Carmelinda Ruggiero, Virginia Boccardi (Università degli Studi di Perugia-Azienda Ospedaliera S.M. della Misericordia, Perugia, Struttura Complessa di Geriatria);
Pietro Minuz, Luigi Fondrieschi, Sarah Morellini, Andrea Sartorio (Azienda Ospedaliera Universitaria Verona, Policlinico GB Rossi, Verona, Medicina Generale per lo Studio ed il Trattamento dell’Ipertensione Arteriosa);
Mario Pirisi, Gian Paolo Fra, Daniele Sola, Mattia Bellan (Azienda Ospedaliera Universitaria Maggiore della Carità, Novara, Medicina Interna 1);
Emilio Simeone, Rosa Scurti, Fabio Tolloso (Ospedale Civile Santo Spirito di Pescara, Geriatria);
Roberto Tarquini, Alice Valoriani, Silvia Dolenti, Giulia Vannini, Francesco Cei, Marco Gucci, Antonella Fardus Al Refaie, Virginia Morelli, Fabio Salvadori (Ospedale San Giuseppe, Empoli, USL Toscana Centro, Medicina Interna I);
Sergio Harari, Chiara Lonati, Federico Napoli, Italia Aiello, Gabriele Tarallo, Chiara Apostolico (Clinica Medica a indirizzo cardio-respiratorio, Multimedica IRCSS, Milano);
Ferdinando Carlo Sasso, Teresa Salvatore, Lucio Monaco, Carmen Ricozzi, Francesca Coviello, Maria Elena Corona, Giovanni Di Lorenzo, Serafina Schettino, Christian Catalini, Davide Nilo (Policlinico Università della Campania L. Vanvitelli, UOC Medicina Interna);
Ilaria Indiano, Alberto Pilotto, Federica Gandolfo, Davide Gonella, Federica Cacioppo (Ente Ospedaliero Ospedali Galliera Genova, SC Geriatria Dipartimento Cure Geriatriche, Neurologiche e Riabilitazione);
Moreno Tresoldi, Enrica Bozzolo, Sarah Damanti, Gaia Deonette, Giulia Marazzi, Rita Venezia, Lorenzo Ciocca, Jasmin Mahajne, Chiara Calabrese, Giusy Faggiano, Marta Secci, Martina Laffranchi, Ilaria Vergine, Alessandro Marinosci, Cecilia Bussolari, Giovanni Benanti, Francesca Conti, Filippo Sciammetta, Rabia Saeedbhatti (IRCCS Ospedale San Raffaele–Milano, Medicina Generale e delle Cure Avanzate);
Paolo Pasquero, Massimo Porta, Miriam Gino, Stefania Morra Di Cella, Bianca Pari, Edoardo Pace, Alessandro De Salve (AOU Città della Salute e della Scienza di Torino–Torino, Medicina Interna 1U);
Patrizia Tilocca, Sebastiana Maria Atzori (Azienda Ospedaliero Universitaria di Sassari– Sassari, Geriatria);
Alberto Moggi Pignone, Giulia Bandini, Maria Cristina De Santis, Chiara Di Cencio, Anna Lo Cricchio (Azienda Ospedaliero Universitaria Careggi Firenze, Firenze, Medicina Interna 4);
Maria Lorenza Muiesan, Carolina De Ciuceis, Stefano Tenore, Fabio Cherubini, Francesco Russomanno (Spedali Civili di Brescia, Brescia, Medicina Generale 2);
Nicola Liuzzi, Sokol Rrodhe, Alice Parisi, Eleonora Croce (Ospedale Civile “Edoardo Agnelli” Pinerolo-Torino, Medicina Generale);
Fiammetta Pagnozzi, Emanuela Messa, Francesca Gaia Bacchieri (Ospedale Civico di Chivasso ASL Torino 4, Torino, S.C. Medicina Interna);
Gaetano Serviddio, Antonino Davide Romano (Ospedali Riuniti di Foggia, Foggia, UOC Epatologia a Direzione Universitaria);
Gianluigi Vendemiale, Francesco Bellanti, Aurelio Lo Buglio (Azienda Ospedaliero-Universitaria Policlinico Riuniti di Foggia, Foggia, UOC Medicina Interna e dell’Invecchiamento);
Antonino Tuttolomondo, Domenico Di Raimondo, Edoardo Pirera, Riccardo De Rosa (Azienda Ospedaliera Universitaria Policlinico Giaccone Policlinico di Palermo, Palermo, UOC di Medicina Interna con Stroke Care);
Francesca Dassie, Angelo Di Vincenzo, Sara Brandolese (Azienda Ospedaliera Università di Padova, Padova, UOC Clinica Medica III);
Agostino Di Ciaula, Carlo Custodero, Antonino Noto, Chiara Valentina Luglio, Piero Portincasa (Azienda Ospedaliero-Universitaria Policlinico di Bari, Bari, Medicina Interna Universitaria “Augusto Murri”);
Leonardo A. Sechi, Cristiana Catena, Gabriele Brosolo, Stefano Marcante, Andrea Da Porto, Luca Bulfone, Antonio Vacca (Azienda Sanitaria Universitaria Friuli Centrale, Università di Udine, Dipartimento di Medicina, Clinica Medica, Udine);
Angelo Baldassare Cefalù, Antonina Giammanco, Carola Maria Gagliardo (Azienda Ospedaliera Universitaria Policlinico Giaccone Policlinico di Palermo, Palermo, UOC di Astanteria e MCAU);
Elena Campello, Chiara Simion (Azienda Ospedaliera Università di Padova, Padova, Medicina Generale a Indirizzo Trombotico e Emorragico);
Michela Zanetti, Paolo De Colle, Giuseppe Castiglia (Azienda Sanitaria Universitaria Integrata di Trieste, SC(UCO) Geriatria.
