# Supplementary material for: Anticoagulant Therapy in Elderly Hospitalized Patients with Atrial Fibrillation: A Critical Appraisal of Data from the Italian REPOSI Registry
Source: J Clin Med. 2026 Apr 24;15(9):3265. doi: 10.3390/jcm15093265 (PMC13164352; doi:10.3390/jcm15093265)
Supplement: Supplementary file 1 [file jcm-15-03265-s001.zip › jcm-4238906-supplementary.pdf]

Supplementary Materials

Table S1a. Multiple Imputation for the association with change in drug therapy at discharge (2010-2023)

		NO change	YES change	Univariate OR (95% CI)	Age and sex adjusted OR (95% CI)	Multivariate OR (95% CI)	Multivariate p-value
		N=1547	N=514			N=2061	
Age		81.5 (7.1)	82.1 (7.4)	1.01 (1.00-1.02)	1.01 (0.99-1.02)	1.01 (0.99-1.02)	0.48
Sex							
	Female	765 (49.5%)	293 (57.0%)			1	
	Male	782 (50.5%)	221 (43.0%)	0.74 (0.60-0.90)	0.75 (0.61-0.92)	0.80 (0.63-1.01)	0.06
Weight		71.8 (0.40)	70.7 (0.68)	1.00 (0.99-1.00)	1.00 (0.99-1.01)	1.00 (0.99-1.01)	0.74
CIRS Severity at admission		1.75 (0.01)	1.69 (0.01)	0.53 (0.39-0.74)	0.55 (0.39-0.76)	0.54 (0.38-0.78)	0.001***
CHA_2DS_2		4.5 (1.3)	4.5 (1.4)	1.01 (0.94-1.09)	0.95 (0.88-1.03)	1.02 (0.93-1.11)	0.67
Hemoglobin at admission		11.73 (0.06)	11.65 (0.11)	0.98 (0.94-1.03)	0.99 (0.95-1.04)	0.97 (0.93-1.02)	0.30
Platelets at admission		225.68 (2.33)	229.93 (4.10)	1.00 (1.00-1.00)	1.00 (1.00-1.00)	1.00 (1.00-1.00)	0.61
INR at admission		1.82 (0.04)	1.51 (0.06)	0.74 (0.64-0.85)	0.74 (0.65-0.85)	0.75 (0.65-0.85)	<0.001***
eGFR at admission		50.31 (0.66)	51.01 (1.20)	1.00 (1.00-1.01)	1.00 (1.00-1.01)	1.00 (1.00-1.01)	0.43
Admission Year							
	2010-2015	703 (45.4%)	206 (40.0%)			1	
	2016-2023	844 (54.6%)	308 (60.0%)	1.25 (1.02-1.53)	1.24 (1.01-1.52)	1.21 (0.98-1.49)	0.07

* $p < 0.05$, ** $p \leq 0.01$, *** $p \leq 0.001$

Table S1b. Multiple Imputation for the association with change in drug therapy at discharge (2016-2023)

		NO change	YES change	Univariate OR (95% CI)	Age and sex adjusted OR (95% CI)	Multivariate OR (95% CI)	Multivariate p-value
		N=844	N=308			N=1152	
Age		82.2 (7.2)	82.7 (7.5)	1.01 (0.99-1.03)	1.01 (0.99-1.02)	1.01 (0.98-1.03)	0.59
Sex							
	Female	409 (48.5%)	180 (58.4%)			1	
	Male	435 (51.5%)	128 (41.6%)	0.67 (0.51-0.87)	0.68 (0.52-0.89)	0.78 (0.57-1.07)	0.13
Weight		72.2 (0.5)	69.6 (0.8)	0.99 (0.98-1.00)	0.99 (0.98-1.00)	0.99 (0.98-1.00)	0.10
CIRS Severity at admission		1.7 (0.01)	1.7 (0.02)	0.61 (0.40-0.93)	0.61 (0.40-0.93)	0.56 (0.34-0.90)	0.02*
CHA_2DS_2		4.5 (1.3)	4.6 (1.5)	1.04 (0.95-1.15)	0.97 (0.88-1.08)	1.05 (0.93-1.18)	0.43
Hemoglobin at admission		11.6 (0.1)	11.1 (0.1)	0.90 (0.85-0.96)	0.91 (0.86-0.97)	0.89 (0.83-0.94)	<0.001***
Platelets at admission		224.0 (3.1)	231.6 (5.6)	1.00 (1.00-1.00)	1.00 (1.00-1.00)	1.00 (1.00-1.00)	0.73
INR at admission		1.8 (0.04)	1.6 (0.1)	0.84 (0.72-0.97)	0.84 (0.72-0.98)	0.83 (0.72-0.97)	0.02*
eGFR at admission		49.1 (0.8)	49.4 (1.5)	1.00 (0.99-1.01)	1.00 (1.00-1.01)	1.01 (1.00-1.01)	0.10

* $p < 0.05$, ** $p \le 0.01$, *** $p \le 0.001$

Table S2a. Change in drug therapy at discharge (2016-2023): No DOACs at admission to DOACs at discharge

		DOAC no (discharge)	DOAC yes (discharge)	Univariate OR (95% CI)	Age and sex adjusted OR (95% CI)	Multivariate OR (95% CI)	Multivariate p-value
		N = 599	N = 91				
Age		82.8 (7.4)	81.6 (7.7)	0.98 (0.95-1.01)	0.98 (0.95-1.01)	1.01 (0.97-1.05)	0.64
Sex							
	Female	311 (51.9%)	48 (52.7%)			1	
	Male	288 (48.1%)	43 (47.3%)	0.97 (0.62-1.50)	0.92 (0.59-1.44)	0.85 (0.50-1.47)	0.57
Weight		70.6 (0.62)	71.6 (1.6)	1.00 (0.99-1.02)	1.00 (0.99-1.02)	1.00 (0.98-1.01)	0.62
CIRS Severity at admission		1.77 (0.01)	1.63 (0.03)	0.23 (0.10-0.49)	0.22 (0.10-0.48)	0.34 (0.14-0.80)	0.01**
Barthel		72.7 (1.2)	79.7 (2.6)	1.01 (1.00-1.02)	1.01 (1.00-1.02)	1.01 (1.00-1.02)	0.17
CHA_2DS_2		4.6 (1.3)	4.3 (1.5)	0.84 (0.71-1.00)	0.82 (0.68-1.00)	0.98 (0.80-1.21)	0.85
Hemoglobin at admission		11.3 (0.1)	11.9 (0.2)	1.11 (1.00-1.23)	1.11 (1.00-1.23)	1.05 (0.94-1.17)	0.42
Platelets at admission		221.5 (3.7)	227.5 (8.9)	1.00 (1.00-1.00)	1.00 (1.00-1.00)	1.00 (1.00-1.00)	0.52
INR at admission		1.99 (0.1)	1.70 (0.2)	0.84 (0.68-1.04)	0.83 (0.67-1.04)	0.85 (0.69-1.07)	0.16
eGFR at admission		46.18 (1.01)	57.09 (2.9)	1.02 (1.01-1.03)	1.02 (1.01-1.03)	1.02 (1.00-1.03)	0.007**

* $p < 0.05$, ** $p \leq 0.01$, *** $p \leq 0.001$

Table S2b. Change in drug therapy at discharge (2016-2023): VKAs at admission to DOACs at discharge

		DOAC no (discharge)	DOAC yes (discharge)	Univariate OR (95% CI)	Age and sex adjusted OR (95% CI)	Multivariate OR (95% CI)	Multivariate p-value
		N = 300	N = 20				
Age		82.1 (7.2)	80.4 (7.1)	0.97 (0.91-1.03)	0.97 (0.91-1.03)	0.98 (0.91-1.06)	0.63
Sex							
	Female	154 (51.3%)	10 (50.0%)			1	
	Male	146 (48.7%)	10 (50.0%)	1.05 (0.43-2.61)	1.00 (0.40-2.50)	0.88 (0.29-2.65)	0.82
Weight		71.6 (0.9)	75.2 (3.2)	1.02 (0.99-1.05)	1.01 (0.98-1.05)	1.02 (0.98-1.05)	0.35
CIRS Severity at admission		1.76 (0.02)	1.70 (0.1)	0.49 (0.10-2.35)	0.51 (0.11-2.45)	0.64 (0.12-3.52)	0.61
Barthel		75.6 (1.6)	76.3 (5.3)	1.00 (0.98-1.02)	1.00 (0.98-1.02)	1.00 (0.98-1.02)	0.91
CHA_2DS_2		4.7 (1.3)	4.5 (1.3)	0.88 (0.62-1.25)	0.89 (0.60-1.32)	0.95 (0.61-1.46)	0.80
Hemoglobin at admission		11.4 (0.1)	11.8 (0.6)	1.08 (0.88-1.32)	1.07 (0.87-1.31)	1.07 (0.86-1.32)	0.55
Platelets at admission		226.0 (4.9)	254.1 (22.4)	1.00 (1.00-1.01)	1.00 (1.00-1.01)	1.00 (1.00-1.01)	0.20
INR at admission		2.7 (0.1)	3.0 (0.6)	1.09 (0.87-1.36)	1.10 (0.87-1.38)	1.10 (0.86-1.41)	0.44
eGFR at admission		46.8 (1.4)	52.7 (6.5)	1.01 (0.99-1.03)	1.01 (0.99-1.03)	1.00 (0.98-1.02)	0.98

* $p < 0.05$, ** $p \leq 0.01$, *** $p \leq 0.001$
